# Identification and characterization of the MADS-box genes highly expressed in the laticifer cells of *Hevea brasiliensis*

**DOI:** 10.1038/s41598-019-48958-9

**Published:** 2019-09-03

**Authors:** Ying Wang, Di-Feng Zhan, Hui-Liang Li, Dong Guo, Jia-Hong Zhu, Shi-Qing Peng

**Affiliations:** 1 Key Laboratory of Biology and Genetic Resources of Tropical Crops, Ministry of Agriculture, Institute of Tropical Bioscience and Biotechnology, Chinese Academy of Tropical Agricultural Sciences, Haikou, 571101 China; 2 Hainan Academy of Tropical Agricultural Resource, CATAS, Haikou, 571101 China; 3 Institute of Tropical Crops, Hainan University, Haikou, 570228 China

**Keywords:** Plant molecular biology, Secondary metabolism

## Abstract

MADS-box transcription factors possess many functions in plant reproduction and development. However, few MADS-box genes related to secondary metabolites regulation have been identified. In *H*evea *brasiliensis*, natural rubber is a representative *cis*-polyisoprenoids in secondary metabolism which occurs in the rubber laticifer cells, the molecular regulation basis of natural rubber biosynthesis is not clear. Here, a total of 24 MADS-box genes including 4 type I MADS-box genes and 20 type II MADS-box genes were identified in the transcriptome of rubber tree latex. The phylogenetic analysis was performed to clarify the evolutionary relationships of all the 24 rubber tree MADS-box proteins with MADS-box transcription factors from *Arabidopsis thaliana* and *Oryza sativa*. Four type I MADS-box genes were subdivided into Mα (3 genes) and Mβ (1 gene). Twenty type II MADS-box genes were subclassified into MIKC* (8 genes) and MIKC^c^ (12 genes). Eight MADS-box genes (*HblMADS3*, *5*, *6*, *7*, *9*, *13*, *23*, *24*) were predominant expression in laticifers. ABA up-regulated the expression of *HblMADS9*, and the expression of *HblMADS3*, *HblMADS5*, *HblMADS24* were up-regulated by MeJA. The function of HblMADS24 was elucidated. HblMADS24 bound *HbFPS1* promoter in yeast and HblMADS24 activated *HbFPS1* promoter in tobacco plants. Moreover, we proposed that HblMADS24 is a transcription activator of *HbFPS1* which taking part in natural rubber biosynthesis.

## Introduction

MADS-box transcription factors play an indispensable role in plant growth and development^1–4^. In plants, MADS-box genes possess many functions in determination of floral organ identity, floral transition, flowering time determination^3,5–10^, embryo development and seed pigmentation^11^, fruit ripening regulation^12^. The MADS-box gene family is classified into two major categories: type I and type II^13,14^. The type I MADS-box genes are classified into three subgroups including Mα, Mβ and Mγ, whereas the type II MADS-box genes are subdivided into MIKC^C^ and MIKC* types according to different exon and intron structures^4,15,16^. The MIKC^C^ genes are subdivided into 12 clades according to the phylogeny in angiosperms^17^. MADS-box proteins contain a highly conserved MADS-boxdomain which is composed of about 60-amino-acid sequences that bind to CArG box (CC [A/T]_6_ GG)^18–21^. In addition, the type II lineage includes three other domains: the Keratin-like (K) domain, the Intervening (I) domain and the C-terminal (C) region^22–24^. In dicot plants such as *Arabidopsis*, the floral homeotic genes were divided into ABCDE-classes based on function^25–27^. In monocot crops such as rice, two D class genes, OsMADS13 and OsMADS21 are involved in ovule identity and floral meristem^28,29^. Four other MIKC^C^ genes, Suppressor of Overexpression of Constans1 (SOC1)^30,31^, Flowering Locus c (FLC)^32,33^, AGAMOUSLIKE GENE 24 (AGL24)^34,35^ and Short Vegetative Phase (SVP)^36^ played a key role in flower initiation and flowering time. SHATTERPROOF 1–2 and FUL related to fruit ripening^12,26^, TRANSPARENT TESTA16 act in endothelium development and seed pigmentation^11^.

Natural rubber (NR) is a *cis*-1, 4-polyisoprene biopolymer that is obtained commercially from the latex of rubber tree (*Hevea brasiliensis* Muell. Arg)^37^. NR biosynthesis is a branch of the isoprenoid pathway which occurs on the surface of the rubber particle in the rubber laticifer cells^38,39^. NR is biosynthesized by sequential condensations of isopentenyl diphosphates which are synthesized *via* the mevalonate pathway^40–42^. In the rubber tree, farnesyl diphosphate synthase (FPS) is an important enzyme in isoprenoids secondary metabolism. *HbFPS1* expressed obviously in the laticifers which is possible to involve in NR biosynthesis. However, *HbFPS2* and *HbFPS3* have no cell-type specific expression, and they are likely to act as housekeeping nature to involve in isoprenoid biosynthesis^43^.

In *H*. *brasiliensis*, the general NR biosynthesis metabolic pathway is now clear, but the molecular regulation of some NR biosynthesis-related genes is limited^41,44^. To date, few MADS-box genes related to NR biosynthesis have been identified. For instance, three MADS-box genes of *H*. *brasiliensis* were identified. They were differentially expressed in the laticifer cells^45^. HbMADS4 was identified to down-regulate the expression *HbSRPP* involved in NR biosynthesis^46^. In the present study, we identified and systematically analyzed the 24 MADS-box family genes (named *HblMADS1* to *HblMADS24*) from the rubber tree latex transcriptome. Eight MADS-box genes were identified as predominantly expressed in laticifers. Furthermore, HblMADS24 positively regulated the *HbFPS1* expression.

## Results

### Identification and phylogenetic analysis of the MADS-box genes from *H. brasiliensis*

Our laboratory had obtained the *H*. *brasiliensis* latex transcriptome database by the Illumina HiSeq 2000 method^47^. A total of 36 MADS-box unigenes were obtained by scanning the *H*. *brasiliensis* latex transcriptome database. These MADS-box unigenes were used as queries in BLAST against the local *H*. *brasiliensis* genome database. A total of 24 MADS-box genes were confirmed from *H*. *brasiliensis* genome. These MADS-box genes were designated as *HblMADS1* to *HblMADS24*, respectively (Additional file Table S1). The number of nucleotides of all the 24 MADS-box genes coding domain sequence varied from 522 bp to 1101 bp, the number of amino acids encoded the 24 MADS-box proteins varied from 173 aa to 366 aa, and the predicted relative molecular mass ranged from 20.4 to 41.18 kDa, with protein isoelectric point (pI) in the range of 5.04 to 10.14 (Additional file Table S1).

**Figure 1:**
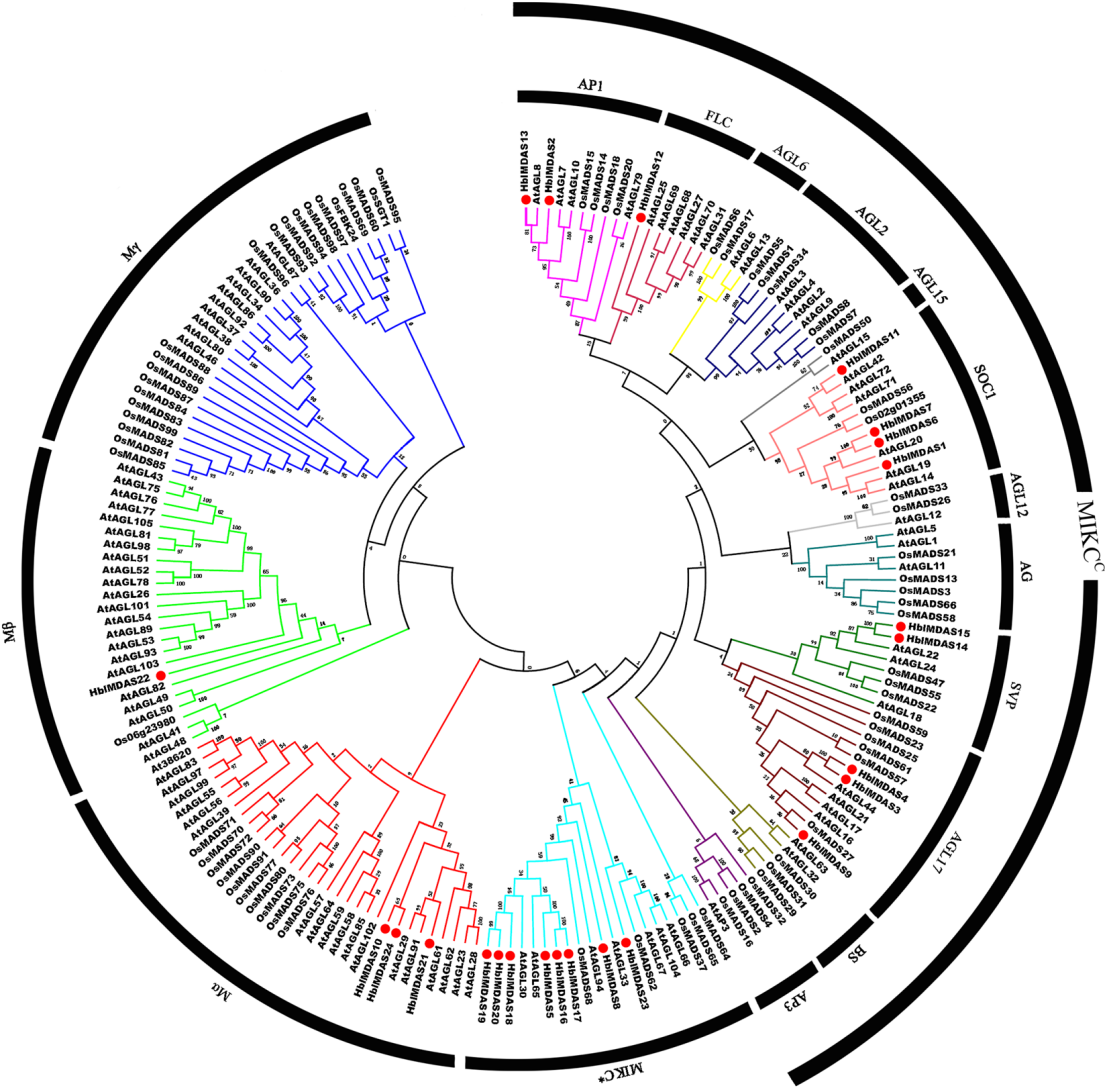
Phylogenetic analysis of the MADS proteins from *H*. *brasiliensis* with *Arabidopsis* and *Oryza sativa* MADS proteins. The phytozome database (https://phytozome.jgi.doe.gov/pz/portal.html) was used to download the *Arabidopsis* and *O*. *sativa* MADS protein sequences. The phylogenetic tree was constructed among all MADS proteins from *H*. *brasiliensis*, *Arabidopsis* and *O*. *sativa* using the neighbor-joining method, and bootstrap analysis were set to 1,000 replications using MEGA6.0. The HblMADS proteins are indicated by red dots. The subgroups are marked by black lines.

To determine the evolutionary relationships between these MADS-box genes in rubber tree latex and other species, the phylogenetic tree was constructed among MADS-box genes from *H*. *brasiliensis* and known MADS-box proteins from *Arabidopsis thaliana* and *Oryza sativa* using the neighbor-joining method (Fig. 1). According to the phylogenetic analysis, 24 MADS-box genes from *H*. *brasiliensis* were classified into two groups, including type I MADS-box genes (4 genes) and type II MADS-box genes (20 genes). Four type I MADS-box genes were subdivided into Mα (3 genes) and Mβ (1 gene). Twenty type II MADS-box genes were subdivided into MIKC* (8 genes) and MIKC^c^ (12 genes). The 12 MIKC^c^ genes were further classified into 5 subfamilies: SOC1 (4 genes), AGL17 (3 genes), SVP (2 genes), AP1 (2 genes) and FLC (1 gene).

### Gene structure analysis and identification of conserved motifs of *H. brasiliensis* MADS-box genes

**Figure 2:**
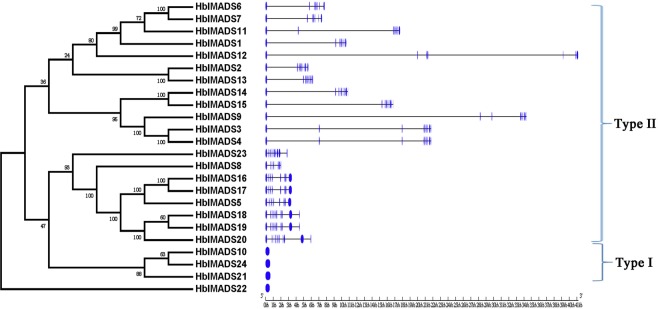
Phylogenetic relationships and Exon-intron structure of the MADS-box genes from *H*. *brasiliensis*. The NJ evolutionary tree was constructed with 1000 bootstrap replicates based on the alignment of full-length amino acid sequences of the MADS-box genes (left side). Meanwhile, the Exon-intron structures of the MADS-box genes are described in the right portion. Exons and introns are represented by blue and black lines. The lengths of the exons and introns of each MADS-box gene are shown proportionally.

The exon/intron patterns of MADS-box genes from *H*. *brasiliensis* were analyzed by the online software GSDS. The genes of the same subfamily had significant similar exon-intron structures, differing only in the number of nucleotides of exon/intron (Fig. 2). In general, the type II MADS-box genes had more exons compared with the type I MADS-box genes, which suggested that the type II MADS-box genes have more complex gene structure. The number of exon of the twenty type II MADS-box genes ranged from 7 to 11, while four type I MADS-box genes contained only one exon. The MIKC* genes displayed less number of nucleotides and more exons than the MIKC^C^ genes.

**Figure 3:**
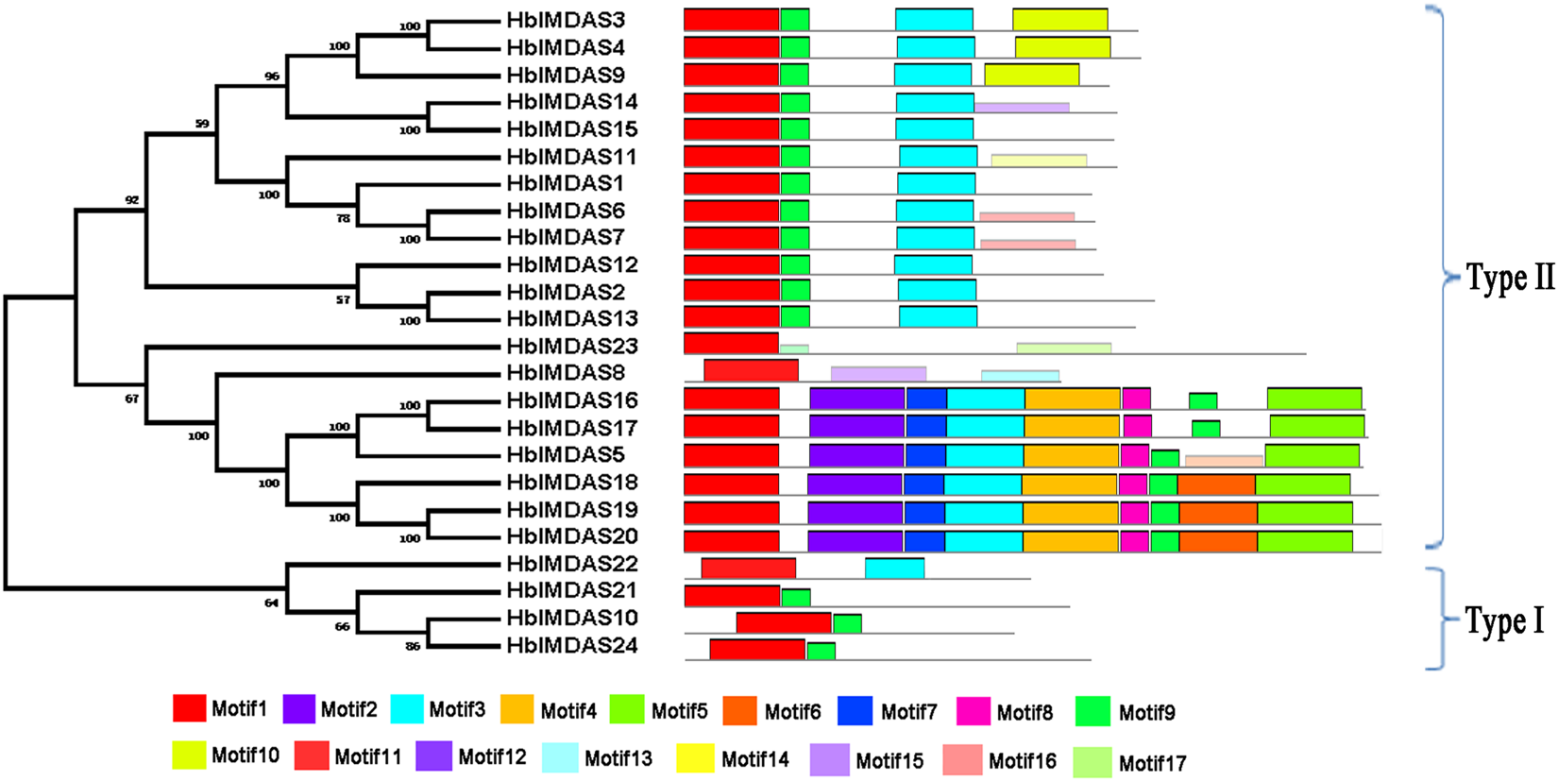
Phylogenetic relationships and conserved motifs of the MADS proteins from *H*. *brasiliensis*. The motif compositions were analyzed using MEME. Motif 1 represented MADS domain, Motif 3 represented K domain.

The conserved motifs of 24 HblMADS proteins were analyzed to illuminate the features of MADS-box protein sequences by MEME motif search tool, resulting in the identification of 17 conserved motifs (Fig. 3). In the same subfamily, most of the closely related proteins distributed similar motif type. All the 24 HblMADS proteins contained motif 1 which is the most typical MADS-box domain in plant MADS-box proteins. Motif 3 represented the most conserved K domain, which was verified in all the type II HblMADS proteins. The K-domain was also only observed in other plants type II MADS-box proteins^48^.

### Expression patterns of MADS-box genes in different tissues of *H. brasiliensis*

**Figure 4:**
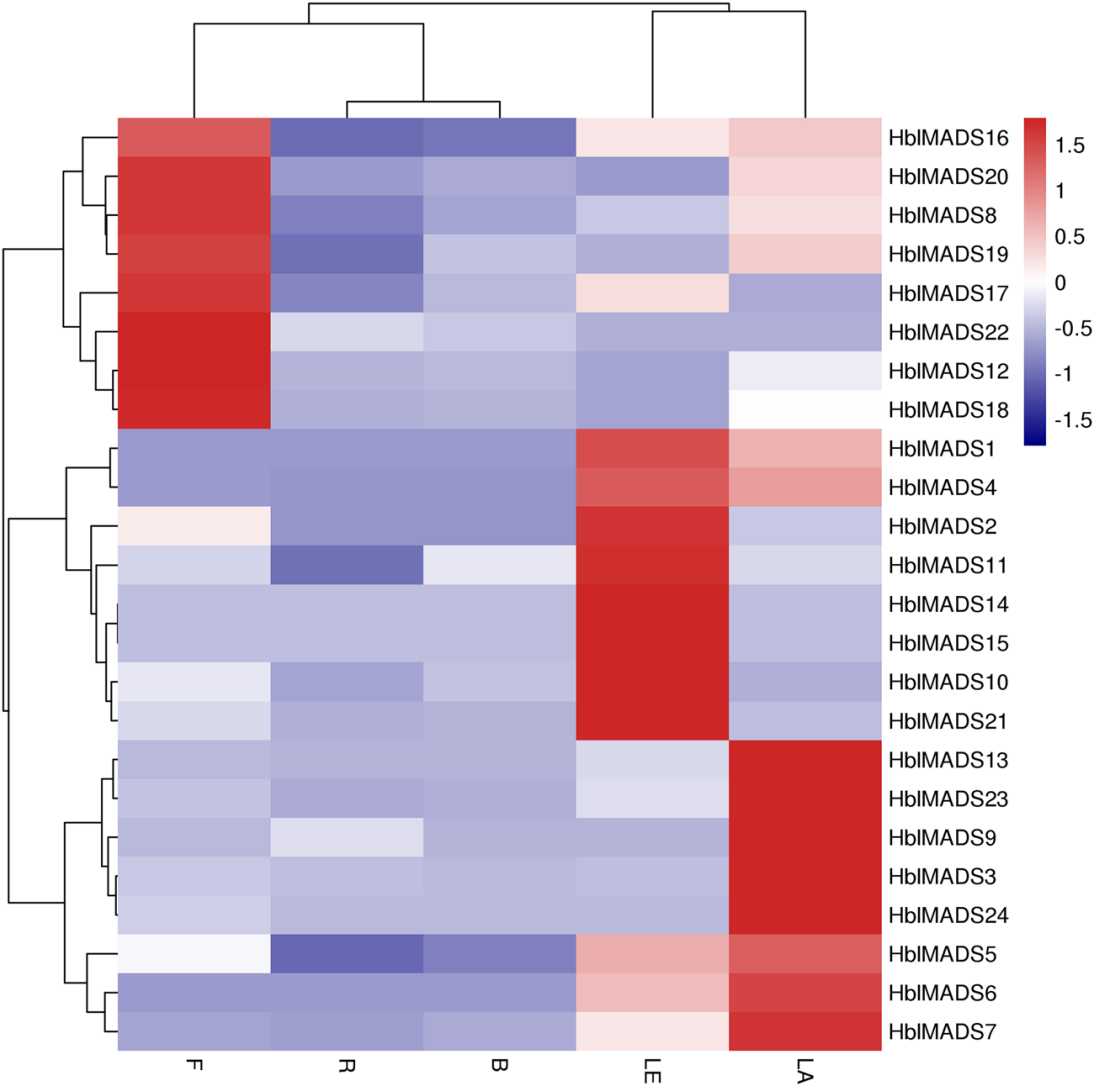
Heat map showing the expression levels of the MADS-box genes in five different tissues of *H*. *brasiliensis*. The heat map was derived from log_2_-based values of three replicates of qRT-PCR data using the online tool (http://www.omicshare. com/tools/Home/Soft/heatmap).The scale represents the relative signal intensity values. Sources of the samples are as follow: R, root; B, bark; LE, leaf; F, flower; LA, latex. The gene expression levels with different color scales are displayed at the top left corner. The groups and subgroups of MADS-box genes are displayed in the right portion.

The expression of all the 24 MADS-box genes was detected in five different tissues (roots, barks, leaves, flowers, latex) by quantitative real-time PCR (qRT-PCR). A heat map showed that different MADS-box genes had diverse expression patterns in different tissues (Fig. 4). Eight MADS-box genes (*HblMADS3*, *5*, *6*, *7*, *9*, *13*, *23*, *24*) had higher expression levels in latex but relatively low expressed in other tissues. Eight MADS-box genes (*HblMADS8*, *12*, *16*, *17*, *18*, *19*, *20*, *22*) maintained significantly high expression level in the flowers, whereas eight MADS-box genes (*HblMADS1*, *2*, *4*, *10*, *11*, *14*, *15*, *21*) in the leaves. By contrast, all MADS-box genes had no expression in the roots and lowly expressed in barks.

### Expression analysis of MADS-box genes in the latex in response to hormone treatments

**Figure 5:**
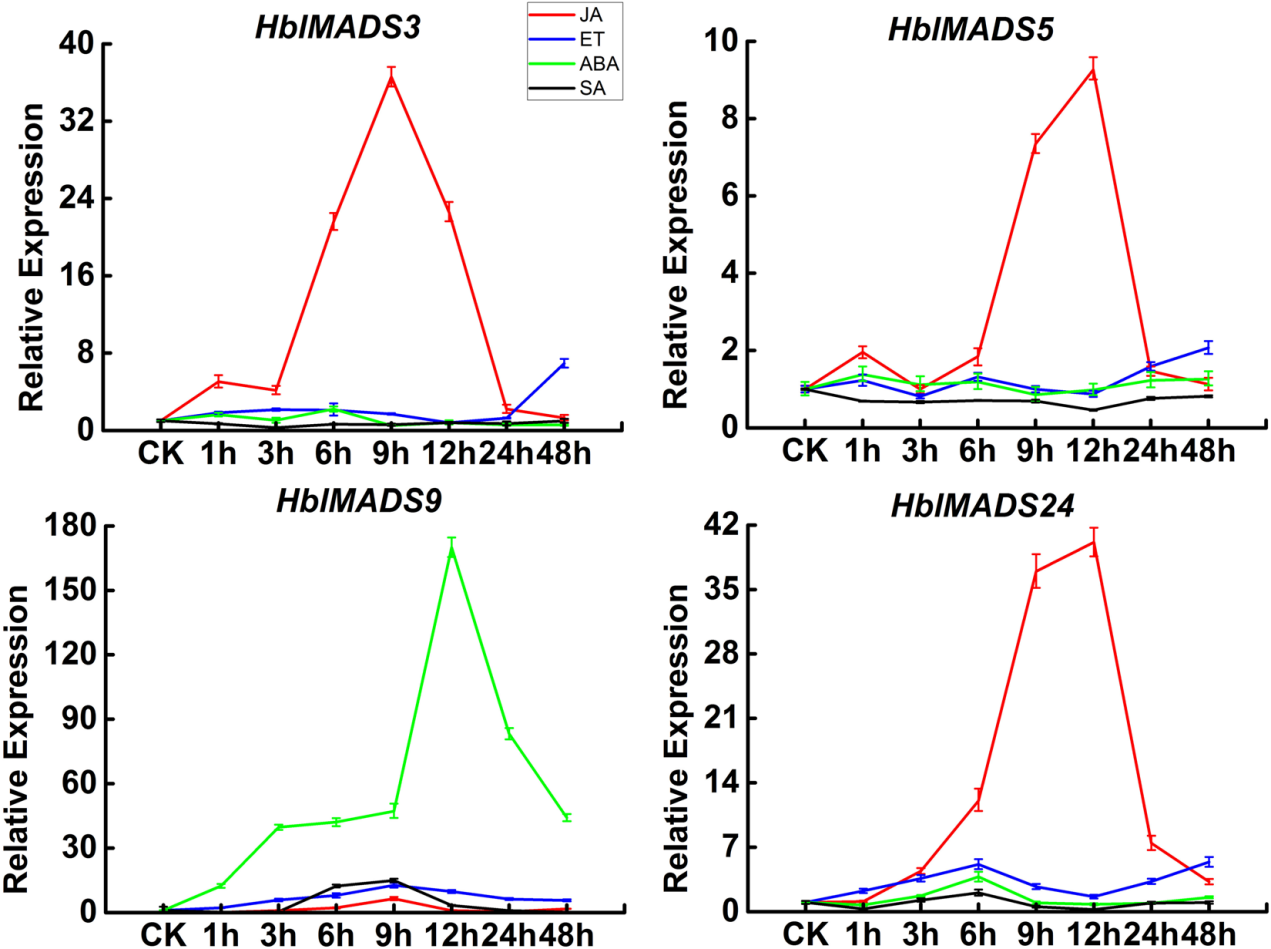
Expression patterns of 4 MADS-box genes responding to phytohormone treatment. RNA extracted from the latex of rubber trees treated with MeJA, ET, ABA, and SA for 0, 1, 3, 6, 9, 12, 24 h and 48 h were subjected to RT-RCR assay. Three independent replicates were calculated to the mean at each time. The standard deviation was indicated with the vertical bars.

Eight MADS-box genes (*HblMADS3*, *5*, *6*, *7*, *9*, *13*, *23*, *24*) maintained higher expression levels in latex. However, *HblMADS6*, *HblMADS7*, *HblMADS13*, *HblMADS23* have been identified^45,46^. *HblMADS3*, *HblMADS5*, *HblMADS9*, *HblMADS24* were analyzed in response to exogenous plant hormones. The expression patterns of these four genes were different under abscisic acid (ABA), ethrel (ET), methyl jasmonate (MeJA), and salicylic acid (SA) treatments (Fig. 5). The results indicated that MeJA treatment up-regulated the expression of *HblMADS3*, *HblMADS5* and *HblMADS24* at either 9 h or 12 h time points, while had no influence on that of *HblMADS9*. ABA treatment evidently up-regulated the expression of *HblMADS9* at 12 h, whereas had not significantly affected that of *HblMADS3*, *HblMADS5* and *HblMADS24*. Under the SA treatment, the expression of *HblMADS9* and *HblMADS24* were slightly up-regulated at 9 h or 6 h time point, while had no significantly effect on that of *HblMADS3* and *HblMADS5*. ET stress had no obvious influence on the expression of four *HblMADS* genes.

### Subcellular localization of HblMADS24

**Figure 6:**
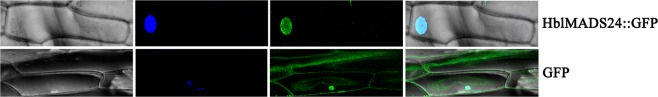
Nuclear localization of HblMADS24. The corresponding bright-field image, DAPI image, fluorescence image, and merged image of HblMADS24-GFP were shown on the upper panel. The corresponding bright-field image, DAPI image, fluorescence image, and merged image of GFP as control were shown on the lower panel.

To elucidate the function of HblMADS24, we performed the subcellular localization analysis on HblMADS24. We found that the green fluorescent protein (GFP) tagged HblMADS24 was indeed present in the nucleus of onion epidermal cells, while GFP alone exhibited a cytoplasmic distribution (Fig. 6).

### Activation of the HbFPS1 promoter by HblMADS24 in yeast

**Figure 7:**
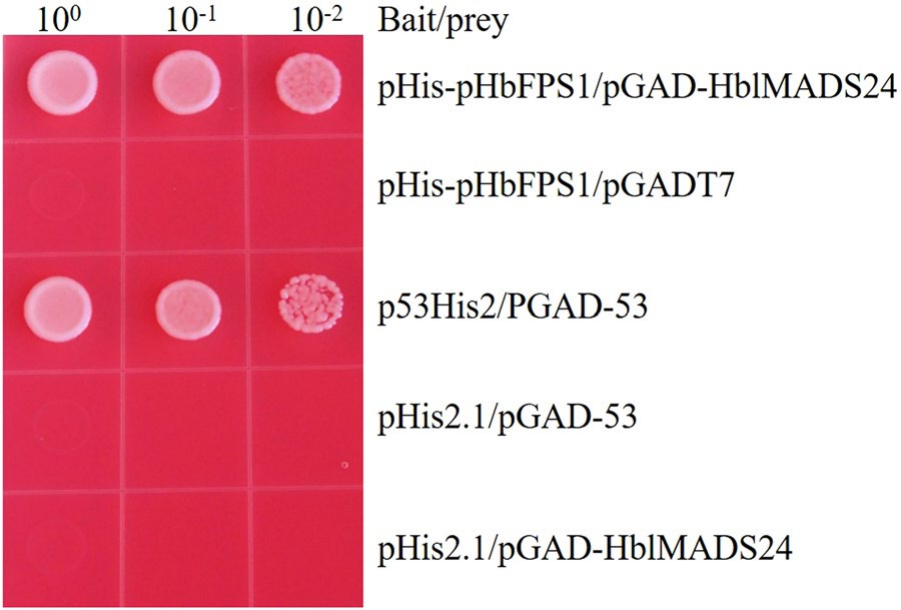
Activation of *HbFPS1* promoter by HblMADS24 in yeast. The yeast clones harboring pGAD-HblMADS24 and pHis-pHbFPS1 could grow on SD/-Trp/-His/-Leu selective medium containing 70 mM 3-AT at 30 °C for 3 days.

*HbFPS1* expressed obviously in the laticifers which is possible to involve in natural rubber biosynthesis^43^. The yeast one-hybrid analysis was performed to investigate whether HblMADS24 binds the *HbFPS1* promoter. The yeast clones harboring pGAD-HblMADS24 and pHis-pHbFPS1 could grow on triple dropout medium lacking histidine, tryptophan, and leucine (SD/-Trp/-His/-Leu) containing 70 mM 3-amino-1, 24-triazole (3-AT), suggesting HblMADS24 bound the *HbFPS1* promoter in yeast (Fig. 7).

### Activation of the HbFPS1 promoter by HblMADS24 in plants

**Figure 8:**
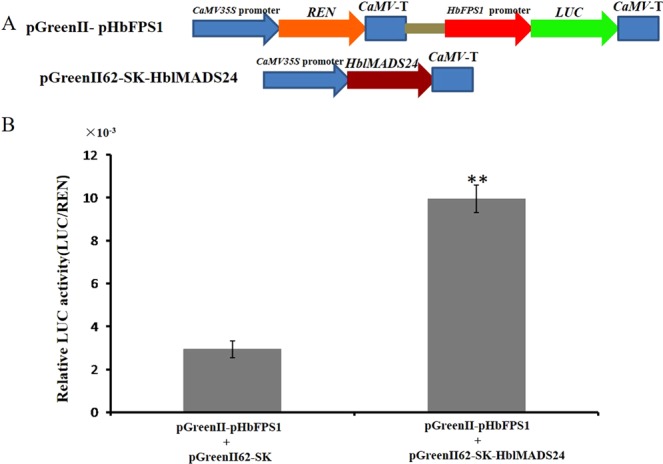
Transient dual-LUC was determined in tobacco leaves. (**A**) The expression vectors used in the transient dual-LUC assays. (**B**) HblMADS24 can activate the *HbFPS1* promoter by transient dual-LUC analysis in tobacco leaves. The values of LUC/REN represent the level of HblMADS24 activation of the *HbFPS1* promoter. The standard deviation was indicated with the error bars. Statistical significance was determined by ANOVA (**P < 0.01).

Since HblMADS24 was able to interact with the *HbFPS1* promoter in yeast, the Dual-LUC method was used to investigate whether HblMADS24 can regulate the *HbFPS1* promoter in plants. The reporter strain pGreen-pHbFPS1 and effector strain pGreenII62Sk-HblMADS24 were mixed and injected into tobacco leaves for Dual-LUC assays. The *HbFPS1* promoter drove luciferase expression weakly alone, while the HblMADS24 expression induced an obvious increase in luciferase activity (Fig. 8). The result indicated that HblMADS24 activated the *HbFPS1* promoter expression.

## Discussion

The MADS-box genes have been identified and characterized in some plants species, such as *Arabidopsis thaliana*^3^, *Raphanus sativus*^9^, *Oryza sativa*^49^, *Populus trichocarpa*^50^, *Zea mays*^51^, *Brassica rapa*^52^, *Vitis vinifera*^53^, *Malus domestica*^54^, *Prunus mume*^55^. *H*. *brasiliensis* is a well-known rubber-producing plant used to produce commercially valuable natural rubber^56^. However, the characterization and systematically analysis of the MADS-box genes family has not been performed in *H*. *brasiliensis*.

In this study, we comprehensive analyzed 24 MADS-box family genes from the rubber tree latex transcriptome, including phylogenetic analysis, gene structures, conserved motifs distribution, expression patterns in different tissues and in response to hormone treatments, gene functional analysis. 24 MADS-box genes were classified into 8 subgroups, which provided a firm basis for better realizing the function of MADS-box genes. In addition, the function of the protein will probably change because of the different exon/intron patterns^57^. Twenty type II MADS-box genes had 7 to 11 exons, while four type I MADS-box genes had only one exon, which could suggest that the type II MADS-box genes contained more variable and complex function. The similar results had also been observed in *Arabidopsis thaliana*^3^, Chinese cabbage^52^, apple^54^, *Prunus mume*^55^, soybean^58^.

The expression patterns of all the 24 MADS-box genes were detected in five different tissues (roots, barks, leaves, flowers, latex) by qRT-PCR. A heat map showed that most MIKC* genes were highly expressed in flowers, which were consistent with previous studies in *Arabidopsis thaliana* and *Oryza sativa*^14,59^. However, most MIKC^C^ genes were highly expressed in leaf and latex. Type I displayed similar or distinct expression profiles. For instance, *HblMADS24* was more expressed in latex, whereas *HblMADS22* was uniquely expressed in flower, *HblMADS10* and *HblMADS21* was more expressed in leaf, which demonstrated that these genes may have multiple functions.

Phytohormone plays key role in NR biosynthesis^60^. Laticifers differentiation is specifically induced by JA^61^. Moreover, JA up-regulated NR biosynthesis-related genes expression, suggesting NR biosynthesis in laticifers is regulated by JA signalling^60,62–64^. ET has been widely applied to stimulate rubber production^65,66^. ABA treated rubber trees exhibited to increases in latex yield^67^. ABA markedly up-regulated NR biosynthesis-related genes expression, suggesting NR biosynthesis in laticifers is also regulated by ABA signalling^68^. SA could also induce a transient increase latex yield^67^. How these hormones are connected to the NR biosynthesis pathway and how their action is integrated with other regulatory circuits is currently unknown. In our present study, ABA up-regulated the expression of *HblMADS9*. NR biosynthesis was probably regulated by JA signaling in laticifers^69,70^. MeJA regulated the expression of *HblMADS3*, *HblMADS5*, *HblMADS24*, which indicated *HblMADS3*, *HblMADS5*, *HblMADS24* may play an important role in JA signaling pathway. But the regulatory mechanism of NR biosynthesis is not clear^71,72^. It will be of great interest to elucidate whether MADS-box transcription factors can regulate NR biosynthesis with JA and ABA as the regulatory signals.

Compared to type II MADS-box genes, the data about type I MADS-box genes is inadequate^73^. Previous studies indicated that *Arabidopsis* type I MADS-box genes are involved in plant development and reproduction^73–76^. Little information is available on type I MADS-box participating in the regulation of secondary metabolism. In our present study, HblMADS24 is a typical I MADS-box gene that bound the *HbFPS1* promoter. Moreover, HblMADS24 activated the *HbFPS1* promoter, suggesting HblMADS24 maybe participate in the regulation of natural rubber biosynthesis. As a result, over-expression of HbFPS1 driven by HBMADS24 would increase latex yield. Identification of the MADS-box genes highly expressed in *H*. *brasiliensis* laticifers cells would greatly help to uncover the molecular regulation basis of natural rubber biosynthesis.

## Materials and Methods

### Plant materials

*H*. *brasiliensis* (Wenchang11) were cultivated in the Hainan Agricultural Reclamation in Wenchang, Hainan. The shoots of two-year-old rubber trees were treated with 200 μm ABA, 200 μm SA, 0.07% JA and 0.5% ET according to the method previously^61^. For each hormonal treatment, the latex samples were collected and mixed from one group of ten-trees when treated at 1, 3, 6, 9, 12, 24 and 48 h^77^. One group of ten-trees without any treatment was as control. All the samples were quickly stored in the RNA extraction buffer. Four other tissues of rubber tree: roots, barks, leaves, and flowers were sampled for RNA extraction.

### Identification of the MADS-box genes in the laticifer cells

The local *H*. *brasiliensis* genome database had been established using the rubber tree genome data^72,78^ and NCBI-Blast-2.2.28+-win32 software in our previously study^79^. A total of 36 MADS-box unigenes were obtained in the rubber tree latex transcriptome database^47^. The MADS-box unigenes were used as queries in searching for MADS-box genes loci in the local genome of *H*. *brasiliensis* using the BLASTx. Finally, MADS-box genes were obtained in the local *H*. *brasiliensis* genome database according to the method previously^80^. The molecular weight and pI of each HblMADS protein were predicted using ExPASy database (http://web.expasy.org/compute_pi/)^81^.

### Phylogenetic analysis

The MADS-box transcription factor protein sequences from *Arabidopsis* and *O*. *sativa* were downloaded from the phytozome database (https://phytozome.jgi.doe.gov/pz/portal.html)^82^. The phylogenetic tree was constructed among MADS-box proteins from *H*. *brasiliensis* and known MADS proteins from *Arabidopsis* and *O*. *sativa* according to the method previously^80^.

### Gene structure analysis and identification of conserved motif

The online software GSDS (http://gsds.cbi.pku.edu.cn/)^79^ was utilized to reveal the exon-intron structure and coding domain sequences (CDS) of MADS-box genes from *H*. *brasiliensis*. The MEME (http://meme-suite.org/tools/meme)^80^ was employed to analyze the conserved domains of HblMADS proteins.

### Expression analysis of the MADS-box genes

Latex total RNA was extracted as described previously^77^, and total RNAs from roots, barks, leaves, and flowers were isolated according to Li’s method^46^. The first-strand cDNA was synthesized in accordance with the manual of the RevertAid^TM^ First-Strand cDNA Synthesis Kit (Fermentas, Lithuania). The quantitative real-time PCR (qRT-PCR) was performed in accordance with the manual of the SYBR Premix EX Taq Kit (TaKaRa, Japan). The *HbACT7* was amplified as the standard control^24^. The primers of MADS-box genes from *H*. *brasiliensis* for RT-qPCR were listed in Additional file Table S2. The qRT-PCR reaction conditions were as follows: pre-heating at 95 °C for 3 min, followed by 40 cycles of 95 °C for 10 s, 58 °C for 20 s, and 72 °C for 25 s. Three individual biological reactions were replicated. The relative expression levels were analyzed using the Stratagene Mx3005P Real Time Thermal Cycler software (Agilent, America)^79^.

### Subcellular localization of HblMADS24

The CDS of *HblMADS24* was fused in the pCAMBIA1302 vector to generate pHblMADS24-GFP. The amplified primers were listed in Additional file Table S2. The pHblMADS24-GFP or pCAMBIA1302 was individual introduced into onion epidermal cells by *Agrobacterium*-mediated method. The transformed onion epiderm was placed on Murashige Skoog solid medium in darkness at 26 °C. After culturing for 5 h, onion epiderm was observed under a confocal microscope (Zeiss LSM510, Germany).

### Transcriptional activation

The *HbFPS1* promoter with 975 bp nucleotides that was cloned into pHiS2.1 vector (Clontech), generating bait vector pHis-pHbFPS1. The amplified primers based on described previously^83^. The CDS of *HblMADS24* was fused into pGAD7 vector to generate prey vector pGAD-HblMADS24. The amplified primers were listed in Additional file Table S2. The transcriptional activity of HblMADS24 was detected by transforming pHis-pHbFPS1 and pGAD-HblMADS24 into yeast strain Y187 (Clontech). The introduced yeast were cultured on SD medium lacking tryptophan histidine, and leucine (SD/-Trp/-His/-Leu) adding 70 mM 3-AT at 30 °C for 3 d.

### Agrobacterium-mediated transient expression assays

The *HbFPS1* promoter and the pGreenII 0800 vector were fused to generate pGreen-pHbFPS1. The ORF of *HblMADS24* was inserted the pGreenII62SK vector to form pGreenII62Sk- HblMADS24. All constructed plasmids were transformed into *A*. *tumefaciens* strain GV3103. The strain harbouring pGreen-pHbFPS1 mixed with the strain harbouring pGreenII62SK-HblMADS24 at a volume ratio of 1:6. The tobacco leaves were infiltrated with mixed *A*. *tumefaciens*. After 3 days, total proteins were extracted from the injected area of tobacco leaves. The Dual-LUC assay was conducted according to Hellens' method^84^. The activity of the luciferase and REN-Luc were measured in accordance with the manual of the Dual-Luciferase Reporter Assay System (Promega). The binding activity of HblMADS24 to the *HbFPS1* promoter was measured by LUC/REN. Three biological repeats were measured.

## Supplementary information

Supporting Information
