# Supplementary material for: Identification and characterization of the MADS-box genes highly expressed in the laticifer cells of Hevea brasiliensis
Source: Sci Rep. 2019 Sep 3;9:12673. doi: 10.1038/s41598-019-48958-9 (PMC6722073; doi:10.1038/s41598-019-48958-9)
Supplement: Supplementary file 1 — Supporting Information [file 41598_2019_48958_MOESM1_ESM.pdf]

Supporting Information

Identification and characterization of the MADS-box genes highly expressed in the laticifer cells of *Hevea brasiliensis*

Ying Wang[1], Di-Feng Zhan[1,2], Hui-Liang Li[1], Dong Guo[1], Jia-Hong Zhu[1], Shi-Qing Peng[1,2]*

[1] Key Laboratory of Biology and Genetic Resources of Tropical Crops, Ministry of Agriculture, Institute of Tropical Bioscience and Biotechnology, Chinese Academy of Tropical Agricultural Sciences, Haikou, 571101, China. [2] Institute of Tropical Agriculture and Forestry, Hainan University, Haikou 570228, China

* Correspondence and requests for materials should be addressed to S.P. (email: shqpeng@163.com)

Table S1 Physicochemical properties of HblMADS transcription factor

genes	ORF length（bp）	Amino acid count	Protein M.W(kDa)	PI
HblMADS1	645	214	24.77	9.11
HblMADS2	741	246	28.57	9.16
HblMADS3	720	239	27.59	9.25
HblMADS4	723	240	27.54	9.24
HblMADS5	1077	358	40.75	6.38
HblMADS6	654	217	24.99	8.59
HblMADS7	654	217	24.98	8.61
HblMADS8	597	198	22.92	10.14
HblMADS9	678	225	25.84	9.32
HblMADS10	522	173	20.04	7.72
HblMADS11	687	228	26.19	9.2
HblMADS12	693	220	24.99	7.75
HblMADS13	714	237	27.54	9.21
HblMADS14	684	227	25.74	8.51
HblMADS15	681	226	25.43	6.85
HblMADS16	1077	358	40.54	6.72
HblMADS17	1083	360	40.88	8.09
HblMADS18	1101	366	41.18	7.12
HblMADS19	1101	366	41.13	6.77
HblMADS20	1101	366	41.03	6.59
HblMADS21	609	202	22.46	7.72
HblMADS22	549	182	21.28	5.04
HblMADS23	990	329	37.51	5.84
HblMADS24	642	213	24.24	8.32

The sequence of HblMADSes

>HblMADS1
ATGGTGAGAGGAAGGACTCAGATGAAGAGAATAGAAAACGCAACCAGTAGGCAAGTA
ACCTTTTCAAAGAGAAGAAATGGGCTCCTCAAGAAGGCCTTTGAGCTTTCAGTTCTCT
GCGATGCTGAAGTTGCACTTATCATTTTCTCTCCCAGGGGAAAGCTCTATGAGTTCTCC
AGCTGCAGTATTAACAAGACCATAGAGCGGTATCAGAAGAAAGCCAAGGATCTTGGAA
TCAGTATCAAGGCAGTTCAAGAAAACATGCAGAATGGGAAGGAAGATTCGTTTAGCTT
GGCAAAGAAGATCGAGCTTCTTGAAATTTCTAAAAGAAAACTCCTAGGTGATGAATTG

GAACCATGTTCCATTGATGAGCTACAGCAGTTAGAGAATCAGCTGGAGCGAAGCTTAA
CCAGAATCAGGACAAGAAAGAATCAGCTGTTCAGGGAACAGATCGAGAAGCTAAAGG
AAGAGGAGAAAATCCTGATAGAAGAAAATACAAGGTTGCGGGCCAAGTGTGGGTTGC
AACCATTGGACTTATCAACAACACAAGAGAAGCAACAAATAGCAGACGGAGAAAGCA
TGGAGGTGGAGACAGAATTATTTATAGGTCCACGTCCACCGGAGACACGAACTACCCA
AAAACTATAG

>HblMADS2
ATGGGAAGAGGTAGGGTTCAGCTCAAGCGGATCGAGAACAAGATTAGCAGGCAAGTG
ACTTTTTCGAAGAGAAGGACTGGTCTCCTAAAGAAAGCTCATGAGATCTCAGTTCTTT
GTGATGCTGAGGTTGCTTTGATTGTTTTCTCAACCAAAGGGAAGCTCTTTGAGTACTCC
ACTGATTCAAGCATGGAAAGGATCCTGGAAAGATATGAAAGATATTCGTCTGCAGAAC
GACAAGTTGCTAATGACTCTGGACAAGAGGGAAATTTGTCTTTGGAATGCCCCAAGCT
CATGGCTAGGATTGAGATTTTACAGAGAAGCTTAAGGAACTTTGCAGGAGAAGATTTG
GATCCTATAAGTCTAAGAGAACTCCAACACTTGGAACAACAGATTGATAATGGTCTTAA
GCGCGTTCGAGCAAGAAAGAACCAACTCTACCATGAATCCCTTTCAGAGCTGCAGAA
GAAGGATAAAGCATTGCATGAGCAAAACAGCATGTTAAGCAAGCAGCTCAAGCAAAA
TGAGAAGTCCATGGCTGAGCATGCTAACTGGGAGCAGCGAAATCTTGGTCAGAACCCA
ACCTACTTCATGCCACCGCCGATGCTATCACTTCCACCTCTAACCACTGGTAGCACTCT
CCAGACTAGAGAATTCATGAATGAAAAAGAGGAGGCTACGGCTCAGATTCAGCTGAAT
ACCATGCAGATGCCACCTTGGATGCTTCGTCATGTCAATGAATAG

>HblMADS3
ATGGGAAGGGGAAAGATTGTGATTCGCAGAATCGACAATTCGACGAGCAGGCAAGTG
ACTTTCTCGAAAAGGAGAAGTGGGTTGCTTAAGAAGGCTAGGGAGCTCTCAATCCTTT
GTGATGCAGAAGTTGGAGTAATTATCTTCTCCAGCACTGGCAAGCTCTATGATTATGCA
AGCACTAGTATGAACTCAGTTATTGAACGCTACAACAAGCTGAAAGAGGAACAAAATC
AGCTGATGAATCCTGCTTCAGAAATCAAGTTTTGGCAAAGGGAGGCTGCAAGCTTGAG
GAAGGAGTTGCAATACTTACAAGAATACCATAGGCAATTGATGGGAGAAGAACTTTCT
GGTCTGAGTGTCAAAGATCTACAAAATTTAGAAAACCAACTGGAGATGAGCCTGAAA
GGTGTTCGGATGAAAAAGGACCAAATTTTAACCGATGAAATCAAGGAACAGAACAGA
AAGGGAAATCTCACTTATCAAGAAAATCTAAAATTGCACAAGAAAGTTGATCTTCTCT
GTCAAGAAAATGCAGAATTACGTAAGAAGGCTTGGGAAGAAAGGGATGCAAGTGAAG
CAAACAAAAGTTCCCACCCACCATACACTTTAAGCAATGGATATGACTTGCATGGCCCA
ATCCATCTCCAGTTGAGCCAGCCACAGCCTCATAGCAATGAACCACCACCAAAGTCAA
TGAAACTTGGACTACAACTGCAATAA

>HblMADS4
ATGGGAAGGGGAAAGATTGTGATTCGCAGAATCGACAATTCGACGAGCAGGCAAGTG
ACTTTCTCGAAAAGGAGAAGTGGGTTGCTTAAGAAGGCTAGGGAGCTCTCAATCCTTT
GTGATGCAGAAGTTGGAGTAATTATCTTCTCCAGCACTGGCAAGCTCTATGATTATGCA
AGCACTAGTATGAACTCAGTTATTGAACGCTACAACAAGCTGAAAGAGGAACAAAATC
AGCTGATGAATCCTGCTTCAGAAATCAAGGTTCTCTTTCATCAAGCCGCTGCAAGCTTG
AGGAAGGAGTTGCAATACTTACAAGAATACCATAGGCAATTGATGGGAGAAGAACTTT

CTGGTCTGAGTGTCAAAGATCTACAAAATTTAGAAAACCAACTGGAGATGAGCCTGAA
AGGTGTTCGGATGAAAAAGGACCAAATTTTAACCGATGAAATCAAGGAACAGAACAG
AAAGGGAAATCTCACTTATCAAGAAAATCTAAAATTGCACAAGAAAGTTGATCTTCTC
TGTCAAGAAAATGCAGAATTACGTAAGAAGGCTTGGGAAGAAAGGGATGCAAGTGAA
GCAAACAAAAGTTCCCACCCACCATACACTTTAAGCAATGGATATGACTTGCATGGCCC
AATCCATCTCCAGTTGAGCCAGCCACAGCCTCATAGCAATGAACCACCACCAAAGTCA
ATGAAACTTGGACTACAACTGCAATAA

>HblMADS5
ATGGGAAGGGTGAAGCTAAAGATAAAGAGGTTAGAGAGCACCAGCAATCGGCAAGTG
ACCTATTCAAAACGAAGGAATGGAATCTTGAAGAAAGCTAAGGAATTGTCTATATTATG
CGACATAGATATTGTCCTTCTCATGTTTTCTCCAACGGGAAGGCCTACTTTATATCAAGG
AGAGCACAGCAACATTGAAGATGTTATTGCAAAATTTGCTCAATTAACTCCGCAGGAA
AGGGCCAAAAGGAAAGTGGAGAGCCTTGAAGTATTGAAGAAAACCTTCAAGAAGTTG
GACCATGATGTGAATATGCAAGACTTTTTGGGTTCAAGCACTCAAACAGTTGAGGAGT
TGACCAATCAAGTTAAGTTTTTGCAAGCGCAACTTACAGAAATACATAATAGAATGAGC
TATTGGAGTAACCCTGATAAGGTTGATAGCATAGAACATCTTAGTCAGATGGAAGAATC
GCTAAAAGAGTCTATTAACCAAATCCGTCTGCAAAAGGAAAATTTTGGGAGTTGCCAA
CTTGTGCGACTAGAATGCAGTAGCCAGTTTCAGAATGGAATGGATTTACCTTTGATGAT
AAATGGCATGCAAGAGGCCCAACCACTATCATGGCTTCCAAACAATGGCAATCAGCAT
CTGATATTATCCAATGAGCAGAACTTTTTGCCTCAAAGAGATAAAGAATGCTCAACTGA
TGCATCCCTTCCAGGCTATTCTGTTTACTATGATACTGGCAAACATACTGAAATTGGAAA
TCCGAGACCAGTTGATAACATCGGGCAGGGCAGTGCTGTTTTTAGCAATTTAAGTAGTA
ATACATGTTTAAGTGTACAACTTGATGAACAGTTCTCATACCCTCCATTTAGTAGTTTAA
ATTTGCCTGAAGTTAAGAAAATGAAGCCTGAGATGCAGATGAACTCCCAAGGAATCTG
TTCTGTCCATCAAGTTAATAGCAATTTTGAACTTCCTAGATCTATATATGAAAATGGTCAT
CATACTTGGGTTTCTGCATCTGGGCCTTGCAGCATTGCAATGTTTAGTGAGAACCAATAT
CACCAGCAACCAAATTGA

>HblMADS6
ATGGTGAGAGGAAAGACTCAAATGAAGCGCATAGAGAACGCCACAAGCAGGCAAGTC
ACCTTCTCCAAGCGGCGAAATGGGTTGCTGAAGAAGGCCTTTGAGCTGTCGGTTCTTT
GCGATGCGGAGGTTGCCCTTATCGTCTTCTCTCCTAGAGGGAAGCTCTATGAATTTGCA
AACTCTAGCATGCAGGAAACAATTGAACGTTATCGTAGGCATGTGAAAGATAATCAAAT
TGACGAGAAGAAATCAGATGAAAACATGGAGCTACTGAAGACGGAAGCAGCGAACAT
GGTGAAGAAGATAGAGCTCCTTGAAATTTCAAAAAGGAAACTACTGGGAGAAGGTTT
GGATTCCTGCACTGTTGAAGAATTACAGCAAATTGAACAACAGTTGGAGAGGAGCGTA
AGCAGCATCAGAGCAAGAAAGAATCAGGTTTTCAAAGAACAAATTGAGCGACTAAAA
GAAAAGGAGAGCCAATTGGCAGCTGAAAATGCAAGGTTATCTGAGAAGTGTGGTGTC
CAACCATGGGAAGGCTTGAAAGTGGTGGGAGAAACTCGATACTGTGAAGAAAGTAGC
CTAGTTTCAGATGTGGAGACTGAACTGTTCATTGGACTACCAGAAACAAGAACCAAGG
GCCATCCTCCAAGAAACTGA

>HblMADS7

ATGGGGAGAGGAAAGAGTGGAGCTGAAGAGGATAGAGAACAAGATCAACCGTCAGG
TCACCTTCTCCAAAAGGAGGAATGGGTTGTTGAAGAAAGCTTATGAGCTATCTGTGCT
TTGTGATGCTGAAGTTGCCCTTATCATCTTCTCTAGTCGTGGCAAGGTTTATGAATTTGA
AACTCTAGCATGCAGGAAACAATTGAACGTTATCGTAGGCATGTGAAAGATAATCAAAT
TGACGAGAAGAAATCAGATGAAAACATGGAGCTACTGAAGACGGAAGCAGCGAACAT
GGTGAAGAAGATAGAGCTCCTTGAAATTTCAAAAAGGAAACTACTGGGAGAAGGTTT
GGATTCCTGCACTGTTGAAGAATTACAGCAAATTGAACAACAGTTGGAGAGGAGCGTA
AGCAGCATCAGAGCAAGAAAGAATCAGGTTTTCAAAGAACAAATTGAGCGACTAAAA
GAAAAGGAGAGCCAATTGGCAGCTGAAAATGCAAGGTTATCTGAGAAGTGTGGTGTC
CAACCATGGGAAGGCTTGAAAGTGGTGGGAGAAACTCGATACTGTGAAGAAAGTAGC
CTAGTTTCAGATGTGGAGACTGAACTGTTCATTGGACTACCAGAAACAAGAACCAAGG
GCCATCCTCCAAGAAACTGA

>HblMADS8
ATGGGAAGAAAGAAGCTAAGGATATTGAGATTGGAATGCGCCAAGGAGAGGCAACTA
AAATACTCAAGGAGAAAAGTTGGAATTATAAAGTATTCCAAGAGAAAAGTTGGAGTTT
TAAAGAAGGCAAAGGAACTTTCAACACTATGTGATATTGATCTTGCAATTATCATGATG
TTTTCTCCCGCCGGCAAGCCTTCCCTCTACATTGGCCATGACAAAGATCTGAGTACAGT
TTTAGAGAGACTGGCAAATTTGACTATTGAAGAACGAGAACAAAGGAGAATCTACACT
ATGAAGTTACTAAAGAAAGAATACTCAAAGGCCAATCCAGCAGTTGGTACAGGAAATA
TTTTACTTGACATGCACAGAGCTGAAGCAGTGAAATTTGGAAGCTCACAGCTGCAAAG
ACATCAATTGGGAGAACTGAAACAAAAGCTCACAGAAAAGATGAACATTCTAAGGGA
TTGGAAGAATCCTTTCAATGTTGACAACTTAGCACAGATCAAAATCATGGAAGAGCGT
CTCATTGCAACTCTCCATAGCATCAAAAACAGAAAAAAGGTAGCTGAGCACTTGCAGT
CTCAGACTGTTTCATAA

>HblMADS9
ATGGGGAGAGGGAAGATAGCGATAAGGAGGATAGACAATTCGACGAGCAGGCAAGTG
ACGTTCTCGAAGAGAAGAAATGGACTCTTGAAGAAAGCGAAGGAGCTTGCGATCCTC
TGTGACGCGGAAGTTGGAGTCGTAATCTTTTCTAGCACCGGAAAGCTCTACGATTTCTC
TAGCACCAGCATGAAATCAGTGATTGAAAGATACAACAAATCAAAGGAGGAACATCAG
CAGATGGGAAACCCAATATCTGAAGTGAAGTTCTGGCAAAGGGAGGCAGCTATGTTGA
GGCAACAACTGCAAAACTTACAAGAAAATCACCGGCAAATGATGGGTGAAGAGCTCT
CTGGCTTGAGCGTAAAGGAGTTGCAAAATTTGGAGAGTCGACTTGAAATGAGTCTCCG
GGGTGTTCGTATGAAAAGGGACCAAATCTTAATGGATGAAATACAAGAACTAAACAGA
AAGGGAAACTTCCTTCACCAAGAAAATGTGGAACTCTGTAAGAAGATTTACGGAGCA
GGAAATATAAATGGAGTAAACAGAGATTACTTTAGCACCAACAGTCTGGGCATTGAGG
AGGAATCAAATGTGCCTGTCCATCTTCAGCTTAGCCAGCCACAGCAACAGAACTATGAT
ACACCAGCAAGAGCTACAAAATTGGGACTACAATTGCATTAG

>HblMADS10
ATGGAAATCACCGCGGCAGACTCCGATGCGCTGCCGGAGCAATCCCGGAGAAGAGGC
GCTAGGGAGTGGAGAAGAGGCGGTAGGGGGCGGAGAAGAATTGAAATCAAGAAAAT

ACAAGACAGAAGGAGCCTGATCTTGGCTTTCTCAAAGCGTCGCACTGGTCTTTTCAAA
AAAGCCGGCGAAGTATGCAACTTATGTGGTGTAGAAGCTGCCGTCATCACGTTCTCCC
CGGCGGGAAAACCCTACGCTTTTGGTAAACCTTCTGCGGACTCAGTCGTTCGTCGCTAT
CTAACCGAAAGCATAAGCACTGCCTCTACTAGTGAATTATATCTTCCTGCGGAGGAAGA
GGAAGAGGAAGAGGAAGAGGAAGATGTGTTCTGGTGGCAACAGTCGATAGAGGATTT
GGATATGAAGGAGCTTGTACAGTACAAGGCAAGCTTGGAAGGGTTGAGGAGGAATTT
GATTATGAGAATTGAGGAGATGAAGATGCGGAGAGCTGCGACCAGAAATTTTTTTGAG
TGA

>HblMADS11
ATGGTGAGAGGAAAGATTCAGATGAAGAGGATCGAAAATGCCGCAAGCCGACAAGTG
ACCTTCTCGAAGCGGAGAAATGGACTTTTGAAGAAAGCTTATGAGCTATCCGTTCTATG
CGATGCAGAAGTTGCAGTGCTAATCTTTTCACAGAGAGGAAGGCTCTTTGAGTTCTCA
AGCAATGACATGGAAAAGACCATAGAACGGTACCGTAGAAATGCAAAAGATAAGGTG
CAAGCTGACAGCGTTGATACAGAACAACGCATTCAGCAACAAATTAGCGAATCCACAG
AGATGGTGAAGAAGATCGAGCAACTCAAAATTTCGCAGGGGAAGTTTTTGGGACAAG
GTTTGACTTCTTGTTCTGTGGAAGAGCTCCAAGAGATACACAACCAGCTAGAGAAAGG
CTTAAGCAATATCAAAGAAAGGAAGGCTGAGTTGTTCAAGGAGAAGATAGAGCAACT
AAAAGCGCAGGAAAGGCTCTTGCTGGAAGAGAATGCAAGTTTGCGTGTAAAGTGTGG
TGAGAAGCCATGGCCGCATCCAACTCAACAGAAAGAAGCTGTGACATACTTAAGCTTA
AATGGGAAGAATTCAGAGGTGGAGACTGAATTGTCCATTGGTCTTCCAGAACCAAAAA
TGCACTGTTTGTAGTGGTCATTAAAAGCTTGTTCTTATATAAGAAAAATTTGA

>HblMADS12
ATGGGGAGAGGGAAGGTGCAGCTGAAGCGAATCGAGGATAAAAGCTCTAGGCAGGTG
ACATTCTCCAAGAGAAAGGGCGGTTTAATGAAGAAAGCTAGGGAATTATCGGTGCTCT
GCGACGTTGAAATTGCAATGATCATCTTCTCTGGCGGAGGAAAGCTGTATGAGTTTTCG
AGCGGCGACAGTTTGAACAAAATCCTCGAGCGCCATCAGGTTCGTAAAAATGAGCAA
GCTGCAGTCAACAACATTGATGAATCCAAGAATCAAGCAGAAGTTAAAGCTGTCTCTA
CAAATGCTAATCTTCTGCAAACTCTCCAAAGGTATCTTCTAGAGAGTAACATAGAGCAG
CTGAATCTTACCCAGTTCCTGCAACTAGAGGAACAACTAGATTCCATATTAAGACAGAT
CAGACTGAGAAAGACACAGTTGATGCTCGAGGCGGTAACTGCTCTCCAACAGAAGGA
AAAACAGCTAGCAGAAGAAAATAATCTAATGGGAACAGAGATAGCAGCAATTCTCAAT
GAGGGCAACCACTGCAACAATCTGCAGGACCAAGTTTTCCTGGATATGGAGCTAAATA
ACCAAGCTAATAATGGAAGATATACTGATGGTTCAACATCATATATGCATCCTCTTCCAG
GGGCTATGCTTCGTTTCTTTTAGAGGATTAGCCATGACCTTTGACTGCAATAA

>HblMADS13
ATGGGGAGGGGTAGGGTTCAGCTTAAGAGAATTGAGAACAAGATCAATAGGCAAGTG
ACTTTTTCCAAGAGAAGGTCTGGCTTGTTGAAGAAAGCCCATGAGATCTCTGTGCTTT
GTGATGCTGAGGTTGCTTTGATCGTCTTCTCCACCAAAGGGAAGCTCTTTGAATACTCT
ACTGATTCCTGCATGGAAAGGATCCTCGAACGTTATGAGAGATATTCATATGCAGACAG
GCAGCTTGTTGCGACTGGTAGCGAAACAAATGGTAGCTGGACTCTGGAACATGCAAA
GTTGAAGGCTAGGATGGAGGTTTTACAGAGAAACCAAAGGCATTTCATGGGAGAAGAT

CTTGATACCTTAAGCCTCAAAGAGCTTCAGAGTGTGGAGCAGCAGATTGATTCTGCTCT
TAAGCACATAAGGTCAAGAAAGAACCAATTGATGTATGAATCCATTGCAGAGTTGCAG
AAGAAGAGCAAGGCATTGCAGGAGCAAAACAACCAATTTGCAAAGAAGGTCAAGGA
GAAGGAGAAGGAAATAGCTCAGCAGAATCAAAAGGAGCAACAAAATCATATTATAGAT
TCATCTACTGTTCTACCACCACCAATGCAGTCTTTGAACATAAGAGGCAATAGAGATGA
AGATGAAACAACTCCAATGCAAAATCTAGCCAATGCTGTCTTGCCATCTTGGATGATTC
CCTACCTGAACGAGTAA

>HblMADS14
ATGGCTAGAGAGAAGATCAAGATCAAGAAGATTGACAACATCACAGCCAGGCAAGTG
ACCTTCTCTAAGAGGAGACGAGGGCTTTTCAAGAAAGCTGAAGAGCTTTCTGTTCTTT
GCGATGCTGATGTTGCTCTCATCATTTTCTCTGCTACTGGCAAGCTCTTTGAGTATTGCA
GCTCCAGCATGAAGGACATAATTGCAAGGTATAATCTGCATTCCAATAACCTCGACAAA
CTGGACCGACCATCACTTGAATTGGAGTTAGAGAATAATAATCGCGCCCGGTTGACCA
AAGAAGTTGCTGAAAAGACCTATCAACTAAGGCAGATGAGGGGAGAGAATCTGCAAG
GATTAAACCTAGAGGAATTGCAGCAATTGGAAAAACTGCTTGAAGCTGGACTTACGCG
TGTGCTTGAAACTAAGGGAGATAAGATAATGAATGAGATCTCTATGCTTGAAAAGAAG
GGAGCTCAACTACTAGAAGAGAATAAACGGCTAAAACAGAAGATGACTACTTTCTGGT
GCAAAGGGAAAAGGCCAGTGCATTTGGAGTCGGACGTCGCTGTCCAACAGGAAGAA
GGCATGTCATCGGAATCTGTCACTAACATCTGCAGCTGCAGCAGTGGCCCTCCTCTTGG
GGATGATAGCTCCGATACATCTCTCAAACTAGGGCTGCCCTTTTGA

>HblMADS15
ATGGCTAGAGAGAAGATCAAGATCAAGAAGATTGACAACATCACAGCCAGGCAAGTG
ACTTTTTCCAAGAGGAGACGAGGTCTTTTCAAGAAAGCTGAAGAGCTTTCTGTTCTTT
GCGATGCTGATGTTGCTCTCATCATTTTCTCTGCTACTGGCAAGCTGTTTGAGTATTGCA
GCTCCAGTATGAAGGACATAATTGCAAGGCATAATCTGCATTCCAATAAGCTCGATAAA
TTAGACCGACCATCTCTTGAGTTGGAGCTACAGAATACCAATCACGAGCGGTTGAGCA
AGGAGGTGGCTGAAAAGACTTATCAACTCAGGCAGATGAGGGGAGAGGATCTGCAAG
GATTAAACATGGAGGACTTGCAGCAATTGGAAAAAATGCTTGAAACAGGACTTAGCCG
TGTGCTTGAAACTAAAGGAGATAAGATGATGAATGAGATCTCTGCACTTGAAAAGAAG
GGAGCTCTTCTGCTAGAAGAGAATAAAGCGCTAAAACAGAAGATGATGACATTGTGCA
AGGGAAAAAGGCCAGTTCTTTTGGATTCGGATTTGGCTGTCCAGCAGGAAGAAGGCAT
GTCATCAGAATCCGCCACCAACGTATGCAGCTGCAGCAGTGGCCCTCTTGCTGAAGAT
GATAGCTCGGATACTTCTCTCAAACTAGGACTGCCCTTTTGA

>HblMADS16
ATGGGAAGGGTTAAGCTAAAGATAAAGAGGTTAGAAAGCACTAGCAATCGACAAGTG
ACCTATTCAAAACGAAGGAATGGAATCTTGAAGAAAGCTAAGGAATTGTCTATATTATG
CGACATAGAGATTGCCCTTCTGATGTTTTCTCCAACGGGAAGGCCTACATTATTTCAAG
GAGAGCATAGAAGTATTGAAGATGTTATTGCAAAATTTGCTCAATTAACTCCGCAGGAA
AGGGCAAAAAGGAAATTGGAGAGCCTTGAAGCATTGAGGAAAACCTTCAAGAAGTTG
GACCACGATGTAAATGTACCAGACTTTTTGGGTGCAAGAACTCAGACAGTTGAGGAGT
TGAGTGATCAAGTTAGGTTGTTGCAAGCACAACTTACTGAAATACAGAATAGAATGAG

CTATTGGAGTAACCCTGATAAGGTTGATAGCATAGAACATCTTAGTCAGATGGAAGACT
CGCTAAAAGAGTCAATTATCCAAATCCGTTTGCAAAAGGAGAATTTTGGAATGTGCCA
ACTTATGCCTCTAGAATGCAATAGCCAGTTTCAAAATGGAATGGCTTTACCTTTGATGAT
GAATGGAATGCAAAAGGCTCAACCATTATCATGGCTTCCAAACAATGGAAATCAACAT
CTGATATTGTCCAACGAGCAAAACATTTCCCCCCAAAGAGATATAGAATGCTCAACCAG
TGCGTCCCTACCAGGCTATTCTGGTTACTATGATACTGACAAACATGTTGAAATTGGAAT
TCCAGGAGCACTAGATAACCCAGGGCGGGATGGTGGTGCATTGAGCAATTTAAGTAAC
AATACATGCTTAGGCGTACAAGTCAATGAGCAATTCTCATGCCCTCCATTTAGTAGTTTA
AATTTGCCTGAAGTTAGGAAGATGAAGCCTGAGATGCAGATGAACTCCCAAGGATCCA
ATTATGTTTATCAAGTTAATAGCAATTTTGAACTTCCTAGATCTATATATGAAAATGGTCA
TCATACTTGGGTTTCTGCATCTGGGCCTTGCAGCATTGCAATGTTTAGTGAGAACCAATA
TCACCAGCAACCAAATTGA

>HblMADS17
ATGGGAAGGGTTAAGCTAAAGATAAAGAGGTTAGAAAGCACTAGCAATCGACAAGTG
ACCTATTCAAAACGAAGGAATGGAATCTTGAAGAAAGCTAAGGAATTGTCTATATTATG
CGACATAGAGATTGCCCTTCTGATGTTTTCTCCAACGGGAAGGCCTACATTATTTCAAG
GAGAGCATAGAAGTATTGAAGATGTTATTGCAAAATTTGCTCAATTAACTCCGCAGGAA
AGGGCAAAAAGGAAATTGGAGAGCCTTGAAGCATTGAGGAAAACCTTCAAGAAGTTG
GACCACGATGTAAATGTACCAGACTTTTTGGGTGCAAGAACTCAGACAGTTGAGGAGT
TGAGTGATCAAGTTAGGTTGTTGCAAGCACAACTTACTGAAATACAGAATAGAATGAG
CTATTGGAGTAACCCTGATAAGGTTGATAGCATAGAACATCTTAGTCAGATGGAAGACT
CGCTAAAAGAGTCAATTATCCAAATCCGTTTGCAAAAGGAGAATTTTGGAATGTGCCA
ACTTATGCCTCTAGAATGCAATAGCCAGTTTCAAAATGGAATGGCTTTACCTTTGATGAT
GAATGGAATGCAAAAGGCTCAACCATTATCATGGCTTCCAAACAATGGAAATCAACAT
CTGATATTGTCCAACGAGCAAAACATTTCCCCCCAAAGGAGAGATAAAGAATGCTCAA
CTGATGCATCCCTTCCAGGCTATTCTGTTTACTATGATACTGGCAAACATACTGAAATTG
GAAATCCGAGAGGAGCACTAGATAACCCAGGGCGGGATGGTGGTGCATTGAGCAATTT
AAGTAACAATACATGCTTAGGCGTACAAGTCAATGAGCAATTCTCATGCCCTCCATTTA
GTAGTTTAAATTTGCCTGAAGTTAGGAAGATGAAGCCTGAGATGCAGATGAACTCCCA
AGGATCCAATTATGTTTATCAAGTTAATAGCAATTTTGAACTTCCTAGATCTATATATGAA
AATGGTCATCATACTTGGGTTTCTGCATCTGGGCCTTGCAGCATTGCAATGTTTAGTGA
GAACCAATATCACCAGCAACCAAATTGA

>HblMADS18
ATGGGAAGGGTGAAGCTAAAGATAAAGAGGTTAGAGAGCACCAGCAATCGGCAAGTG
ACCTATGGCAAAAGGAAGCATGGAATCATGAAGAAGGCTAAAGAGTTATCTATTCTATG
TGACATAGATATTATCCTTCTCATGTTCTCACCAACTGGCAAGCCCTCAATATGCAAAGG
AAAGCGGAGCATTGAAGAGGTCATTGCAAAATTTGCTCAGTTAACACCTCAAGAAAG
GGCAAAAAGGAAGTTGGAAAGCCTTGAAGCGCTGAAGAAAACTTTTAAGAAGTTGGA
CCATGATGTTAACATACCTGAATTTCTGGGCACAAGTTCTCAAACAATGGAGGACCTGT
CAAACCAATCGAGGTTATTGCAGAATCAACTTTCTGAAGTGCATAAAAGACTGAGCTAT
TGGACTAATCCAGATAAAATTAACAACATAGAACATCTGGGTCAATTGGAAAATTCACT
CAGGGAATCGCTTAATAAAATTCAAGCTCATAAGGAATATTTAGGAAAACAGCAACTTA

TGTCGCTAGAATGCAATAGTCAGTTTCAAAATGGGATGCATGTACCTTTCAGAATTGGT
GCAGAGCAGCAGCTCCCACCCATGCCATGGATTCCTAATAATGACAGTCAACAGATTGT
GTTACCAGAGGATCCAAATCTACTTCCCAATAGGGATGTCGAATGTTCTGCAAGTTCCT
CCTTTGGAAGTTATTCTGGTTACTTTGGCACAGGAAAAAGTTCCGAGCTATCTAATTCC
AGTCAAGAAAATGGTGTGAATGGTATTCTTAATGAGTTAAGTGGAACTGCATCACTGA
GGCTGCAGTTGGCTGGGCAGTATCCTTACATGCCATACAATCTGAATTTGCTGAATGAT
ACAAAATTCCAATCTGCAGCAGAGATGAACCTACAAGAAAGCCCTGTGGATTTTCATG
TTAATGGAAGTTTTGAAGCTCCCAAACCTGGATACGACGCTAACCCTGGTAGTTGGGC
TTCTTCATCAGGACCATGTGCTGTTACCATGTTTGATGAGCATTTGTATTCCCAGAAAGA
TCATCTGTATGGGAGCAACAATGAGGAATACTACAGAATTTCCTGA

>HblMADS19
ATGGGTAGGGTTAAGCTAAAGATCAAGAAATTGGAGAACACAAATGGCCGTCAAGCG
ACTTATGGCAAAAGGAAACATGGCATCATGAAGAAGGCAAAGGAGTTGTCTATTCTAT
GTGACATAGATATTATCCTTCTCATGTTCTCACCAACTGGCAAGCCTTCAATATGCAAAG
GAAAACGGAGCATTGAAGAAGTCATTGCAAAATTTGCTCAGTTAACACCTCAAGAAA
GGGCAAAAAGGAAGTTAGAAAGCCTTGAAGTGCTGAAGAAAACTTTTAAGAAGTTGG
ACCATGATGTTAACATACCTGAATTTCTGGGCACAAGTTCTCAAACAGTAGAGGACCTG
TCAAACCAATCGAGGTTATTGCAGAATCAACTTTCTGAAGTGCATAAAAGACTGAGCTA
TTGGACTAATCCAGATAAAATTAACAACATAGAACATCTGGGTCAATTGGAAAATTCAC
TCAGGGAATCGCTTAATAAAATTCAAGCTCATAAGGAATATTTAGGAAAACAGCAACTT
ATGTCGCTAGAATGCAATAGTCAGTTTCAAAATGGGATGCATGTACCTTTCAGAATTGG
TGCAGAGCAGCAGCTCCCACCCATGCCATGGATTCCTAATAATGACAGTCAACAGATTG
TGTTACCAGAGGATCCAAATCTACTTCCCAATAGGGATGTCGAATGTTCTGCAAGTTCC
TCCTTTGGAAGTTATTCTGGTTACTTTGGCACAGGAAAAAGTTCCGAGCTATCTAATTC
CAGTCAAGAAAATGGTGTGAATGGTATTCTTAATGAGTTAAGTGGAACTGCATCACTGA
GGCTGCAGTTGGCTGGGCAGTATCCTTACTTGCCAAACAATCTGAATTTGCTGAATGAT
GAAAAATTCCAACCTGCAGCAGAGATGAACCTACAAGAAAGCCCTGTGGATTTTCATG
TTAATGGAAGTTTTGAAGCTCCCAAACCTGGATACGACGCTAACCCTGGTAGTTGGGC
TTCTTCATCAGGACCATGTGCTGTTACCATGTTTGATGAGCATTTGTATTCCCAGAAAGA
TCATCTGTATGGGAGCAACAATGAGGAATACTACAGAATTTCCTGA

>HblMADS20
ATGGGAAGGGTTAAGCTAAAGATAAAGAGGTTAGAAAGCACTAGCAATCGACAAGTG
ACCTATTCAAAACGAAGGAATGGAATCTTGAAGAAAGCTAAGGAATTGTCTATATTATG
CGACATAGAGATTGCCCTTCTGATGTTTTCTCCAACGGGAAGGCCTACATTATTTCAAG
GAAAACGGAGCATTGAAGAAGTCATTGCAAAATTTGCTCAGTTAACACCTCAAGAAA
GGGCAAAAAGGAAGTTAGAAAGCCTTGAAGTGCTGAAGAAAACTTTTAAGAAGTTGG
ACCATGATGTTAACATACCTGAATTTCTGGGCACAAGTTCTCAAACAGTAGAGGACCTG
TCAAACCAATCGAGGTTATTGCAGAATCAACTTTCTGAAGTGCATAAAAGACTGAGCTA
TTGGACTAATCCAGATAAAATTAACAACATAGAACATCTGGGTCAATTGGAAAATTCAC
TCAGGGAATCGCTTAATAAAATTCAAGCTCATAAGGAATATTTAGGAAAACAGCAACTT
ATGTCGCTAGAATGCAATAGTCAGTTTCAAAATGGGATGCATGTACCTTTCAGAATTGG
TGCAGAGCAGCAGCTCCCACCCATGCCATGGATTCCTAATAATGACAGTCAACAGATTG

TGTTACCAGAGGATCCAAATCTACTTCCCAATAGGGATGTCGAATGTTCTGCAAGTTCC
TCCTTTGGAAGTTATTCTGGTTACTTTGGCACAGGAAAAAGTTCCGAGCTATCTAATTC
CAGTCAAGAAAATGGTGTGAATGGTATTCTTAATGAGTTAAGTGGAACTGCATCACTGA
GGCTGCAGTTGGCTGGGCAGTATCCTTACTTGCCAAACAATCTGAATTTGCTGAATGAT
GAAAAATTCCAACCTGCAGCAGAGATGAACCTACAAGAAAGCCCTGTGGATTTTCATG
TTAATGGAAGTTTTGAAGCTCCCAAACCTGGATACGACGCTAACCCTGGTAGTTGGGC
TTCTTCATCAGGACCATGTGCTGTTACCATGTTTGATGAGCATTTGTATTCCCAGAAAGA
TCATCTGTATGGGAGCAACAATGAGGAATACTACAGAATTTCCTGA

>HblMADS21
ATGGGTCGTAAGAAGACTCAAATGAAGATGATTCAGGGAGGGAATGCTCGGCAGGTTT
CTTTTTCAAAGCGCCGATCTGGCATCTTCAAGAAGGCCAGCGAACTTTGTACCTTATGC
ACGGTTGAAACTGCACTTGTTGTCTTCTCCCCTGGTGGCAAGGCCTTCTCCTTTGGCCA
TCCCTGCTTTGAAGCGATTATGAAGAGGCTTGCTGATGCTGAACCAAGGAATCGGGAT
CCTGACTTTGCTCAGCATATGGCTGAGCATGAGGCCACTCTTAGTGAGCTTAACAAACA
GTATTCAGATGCGCTGAAAGAACTGGAAGCTGAAAAGAAGCGAGGAAAGGAGCTAAA
GCAAATGAGAGAGGCTCAGGGCATGCCATTGTTGGATAAACCAATTGAGGAACTCAAC
TTGGACGAGCTGGAGACATTGAAAGCTTTCTTGGAACAAGTCAAGGGAAATCTACTCA
AGCGACTTGCGGAGCTTTCAGTCCAGACTTCGAATCTGTCTGCATCCTCTGAGAATTCT
GCTGAAGCCATTGATCTTCGTGTTACTAACCCAAAAGGAGTTGGTGCTAATACTCATGA
CCATGGTATTAGCTATGAAAATTGA

>HblMADS22
ATGAATCGGAACAACGGGCCGTTGACTCCTCGCAAAGCCATCTTCAGGAAGAGAAAG
GAAACATTGAAGAAGAAAGCAGAAGAGCTCTCCTCCCTGTGCGGTGTTCCAGTTTGC
CTAATCTGTTATCAACCAGATGGGAAGATCGATACATGGCCCGAAGATAAGAAGGAGG
TTGATGATATTCTCATGAAATATACACGTGAAGAAAACATAAACCTGCAGCCTGCAGTA
GGGTTTTTGGACGCAGAGAACAACAATCAAGCTTCTGTGGTGATCAAGAACGACAAC
AGGAAAGAAAAGAAGGAGAAGAAGAAGAAGAAAGTGTTTGAAACATGGGACACCA
GGCTTGATAACTTACCTGAGGAATCTTTAATGGATATTTTGACGGTGTTGGAGGAGAAG
GAAGAGATTTTGGAGGAAAGAATATTGCAGGTAGATAAGTCTAATTCCTATCCTGATTC
TAATAGTAATACAGAAGATGATAACGATGATTTTAGGATTTTAGCAAGAAAATCCAACC
AAGAAACCTTAGAGTTTTTGCAACTTTAG

>HblMADS23
ATGGGAAGAGTGAAGCTTCAGATCAAGAGAATTGAAAATACAACTAACAGGCAAGTT
ACTTACTCCAAAAGAAGAAATGGGCTTATCAAGAAAGCTTATGAACTTTCTGTTCTCTG
CGATGTTGATGTAGCTCTCATCATGTTCTCTCCATCTGGGAGACTCAGTCTCTTTTCTGG
AAACAAAAGCATTGAGGAAATTCTGACGAGATATGTGAATCTTCCTGAGCATGAACGA
GGAAGGCTACATAAGCAAGAGTTCCTAGAAAAGGCTCTTGGTAAGTTAAAAGCTGAA
GGCGATCGAAATCATCAAGCGGCCAGCAACCCCGCGATCACTGATTCTCAGCTGGAGG
AGTTTCAACAAGAAATTGTTAGATTCAAATCCCAAGTGGAGGATATGGAAAAACAAAT
AAGGATATTGGAGGGCAATCTTTCCCATACCACAACATTACCTGAGGCTGAGTACCAAG
AACAGATACTTGAAGAGGCATTGAAGCGCGTGCAGATGCGTAAGCAAGTTTTAGAAGA

AAAGTACAATTCTTCTGGTGCGCCAACAGTTTCACAGTGTAATCAGGCACATCTTCCTC
CAAAGAATGCATATGTAAATGATCTTGTCACAGAAGAAAGTCCAAACAATGTCTTGGAT
TGGCTTCGTCAGAGAGAGCCACAAGTTCAGATCTTGAATTTTTTGGACTCCAATGGCC
TTCTTCCCTTAAGAAACCAGGTGCAGCACGCAGCTGAAATATTCTTACCATCACCTGCA
ACTCTTCTTCATGGACAAAACATAAATCTTGATGATCACATAAGCCCAAGAAGTGGTTT
GGAGGACGACAATAATGTACAACGTCCTGAGTTTGGACAAGTTATTGATGTCAACTTGT
CCCCATGGGCTGAATTTTACCCTACAGGTAACAGCTCAATTCTCACTTCACAACCCAGA
GAAAGAGCTCTTCTTGAATTGTATTTGTCTCGGATTGCACCATCAACCATTTGA

>HblMADS24
ATGGATGGAACGACCGACCAACGGCCGATCAAGAAAAGCAGAGGCCGCCAAAAGATT
GAGATCAAGAAAGTGGAGAAAGAAAACAGTCGCTATGTTACGTTTTCCAAACGTAAA
AATGGGATTTTCAAGAAAGCCACTGAGTTATCTACCTTATGTGGGGCGGATGTAGCTGT
GATTCTTTTCTCCGAACATGGGAAAGTGTTTTCTTGCGGCAACCCGAATGTCGAAAAG
GTTCTTGATCGATATCTTGCTGAAAAGGAAGAGAAATCTTGCATACTGGAAATCGGCAG
CAATGGCAGTGGTGTGACTCAGACGACTTTGCAAGAGCGAGATTACAAGAAATCTCTT
AGCCGGTTGGAAGAGATGAAAAGAGCTTTTACGATGATAAGCAAAAATAGCAACATGA
ACAAGGGTGAATTTTGGTGGGATCTACCGATCGATAACATGGAGAAGGAAGAGCTTGA
AAGTTATAGGGAATCTCTGGAAGAGTTGAAAAAGAATGTAATGACAAGGATTGAGGGG
ATGGCTGCCCATAATGCTGCGGGTGAGTCTAGTATTATTAACCAGTTCATTAATCATAAT
GGGGTTTGGAATAATTCTATTGTTACTTATGATGCTGGTTTGAATAATGGGTTTCTTTAA

Table S2. Primers used in this study

Primers purpose	Gene	Forward primer（5’-3’）	Reverse primer（5’-3’）
qRT-PCR primer	HblMADS1	CATGGAGGTGGAGACAGAATTA	GATGAGCTTGGTATTTAAGTGAAAC
	HblMADS2	TCAGCTGAATACCATGCAGA	CCTTGATGGCTATCTGTCAGT
	HblMADS3	TAGCAATGAACCACCACCAA	GCTGGCAACTGCTTTGTTATT
	HblMADS4	AGCCTCATAGCAATGAACCAC	GCTGGCAACTGCTTTGTTATT
	HblMADS5	ACTTGGGTTTCTGCATCTGG	CAACAATCACTCCTCCCATCAG
	HblMADS6	GTAGCCTAGTTTCAGATGTGGAG	CGTCAATGAAGTCCCTGTCAA
	HblMADS7	TTCAGATGTGGAGACTGAACTG	CAGTTGCCCTTATTGTTGCTTG
	HblMADS8	CGTCTCATTGCAACTCTCCATAG	GAGAGGGAGTTGATGCATGATAG
	HblMADS9	GAATCAAATGTGCCTGTCCATC	CGCATAACATCCATTTCCAGTG
	HblMADS10	TTCGTCGCTATCTAACCGAAAG	CACCAGAACACATCTTCCTCTT
	HblMADS11	GGAAGAATTCAGAGGTGGAGAC	TTTAATGACCACTACAAACAGTGC
	HblMADS12	AGAGGATTAGCCATGACCTTTG	TACAAACTCCACTGCTCTGTTAG
	HblMADS13	CCAATGCTGTCTTGCCATCT	CCCACATCTTACTACGTGCAAC
	HblMADS14	GGATGATAGCTCCGATACATCTC	CCTGGCTTTATTCCTTGTGATAAT
	HblMADS15	GCCCTCTTGCTGAAGATGATA	ACTACTTTGTCTTCCCACCATC
	HblMADS16	GGTCATCATACTTGGGTTTCTG	GTTGCTGGTGATATTGGTTCTC
	HblMADS17	ACTTGGGTTTCTGCATCTGG	CAACAATCACTCCTCCCATCAG
	HblMADS18	TCTGTATGGGAGCAACAATGAG	TCCATGAGACAATCACACTTCC
	HblMADS19	GGGAGCAACAATGAGGAATACT	GTGTGATATCCTACTCCATGAGAC
	HblMADS20	TCTGTATGGGAGCAACAATGAG	TCCATGAGACAATCACACTTCC
	HblMADS21	CTTCGAATCTGTCTGCATCCT	CATGAGTATTAGCACCAACTCCT
	HblMADS22	CCAGGCTTGATAACTTACCTGAG	ACCTGCAATATTCTTTCCTCCA
	HblMADS23	GTGGTTTGGAGGACGACAATA	CTCTGGGTTGTGAAGTGAGAAT
	HblMADS24	TGCGGGTGAGTCTAGTATTATT	ACCCATTATTCAAACCAGCATC
	HbACT7	TGTCAGCAACTGGGACGATATGG	GAGTCATCTTCTCTCTGTT GGC

Subcellular localization primer	pc1302-HblMADS24	CCCATGGGTATGGATGGAACGACCGACCAACG	GGACTAGTTTAAAGAAACCCATTATTCAAACC
Transactivation Assay primer	pHis-HbFPS1	CACTAGTCTGCATTTTTATGATTAAAAAATAG	GTGCGCAGGATTCAAACGGAGATTAGATAGA
	pGADT7-HblMADS24	CGCCATATGGATGGAACGACCGACCAACG	CCGCTCGAGTTAAAGAAACCCATTATTCA
Dual-Luciferase Assay primer	pGreenⅡ-HbFPS1	ACTCTAGACTGCATTTTTATGATTAAAAAATAGTT	TGTGTCGACGGATTCAAACGGAGATTAGATAGAA
	pGreenⅡ 62Sk-HblMADS24	CGGGATCCATGGATGGAACGACCGACCAACG	CCAAGCTTGGTTAAAGAAACCCATTATTCAAACC
